# RETRACTION: Antileukotriene Therapy by Reducing Tau Phosphorylation Improves Synaptic Integrity and Cognition of P301S Transgenic Mice

**DOI:** 10.1111/acel.70523

**Published:** 2026-04-30

## Abstract

**RETRACTION**: GiannopoulosP. F. , ChiuJ. , and PraticòD. , “Antileukotriene Therapy by Reducing Tau Phosphorylation Improves Synaptic Integrity and Cognition of P301S Transgenic Mice,” Aging Cell 17, no. 3 (2018): e12759, 10.1111/acel.12759 29607621 PMC5946065.

The above article, published online on 01 April 2018 in Wiley Online Library (wileyonlinelibrary.com), has been retracted by agreement between the journal Editor‐in‐Chief, Monty Montano; The Anatomical Society; and John Wiley & Sons Ltd. The retraction has been agreed upon following an investigation into concerns raised by the corresponding author, D. Praticò, and by a third party. This identified image manipulation or duplication within Figures 2C, 3C, 4A, and 4C, as well as with a previously published article by the same author group. As a result, the data and the conclusions are considered unreliable.

D. Praticò agreed to the decision to retract. P. F. Giannopoulos and J. Chiu were informed of the decision to retract but remained unresponsive.

**RETRACTION**: P. F. Giannopoulos , J. Chiu , and D. Praticò , “Antileukotriene Therapy by Reducing Tau Phosphorylation Improves Synaptic Integrity and Cognition of P301S Transgenic Mice,” Aging Cell 17, no. 3 (2018): e12759, 10.1111/acel.12759 29607621 PMC5946065.

The above article, published online on 01 April 2018 in Wiley Online Library (wileyonlinelibrary.com), has been retracted by agreement between the journal Editor‐in‐Chief, Monty Montano; The Anatomical Society; and John Wiley & Sons Ltd. The retraction has been agreed upon following an investigation into concerns raised by the corresponding author, D. Praticò, and by a third party. This identified image manipulation or duplication within Figures 2C, 3C, 4A, and 4C, as well as with a previously published article by the same author group. As a result, the data and the conclusions are considered unreliable.

D. Praticò agreed to the decision to retract. P. F. Giannopoulos and J. Chiu were informed of the decision to retract but remained unresponsive.

